# Supplementary material for: The causal role of circulating inflammatory markers in osteoporosis: a bidirectional Mendelian randomized study
Source: Front Immunol. 2024 Jul 18;15:1412298. doi: 10.3389/fimmu.2024.1412298 (PMC11291241; doi:10.3389/fimmu.2024.1412298)
Supplement: Supplementary file 1 [file DataSheet_1.zip › supplementary material/OSTEOPOROSIS-CIM/GCST90274804.tsv.gz_leave_one_out.pdf]

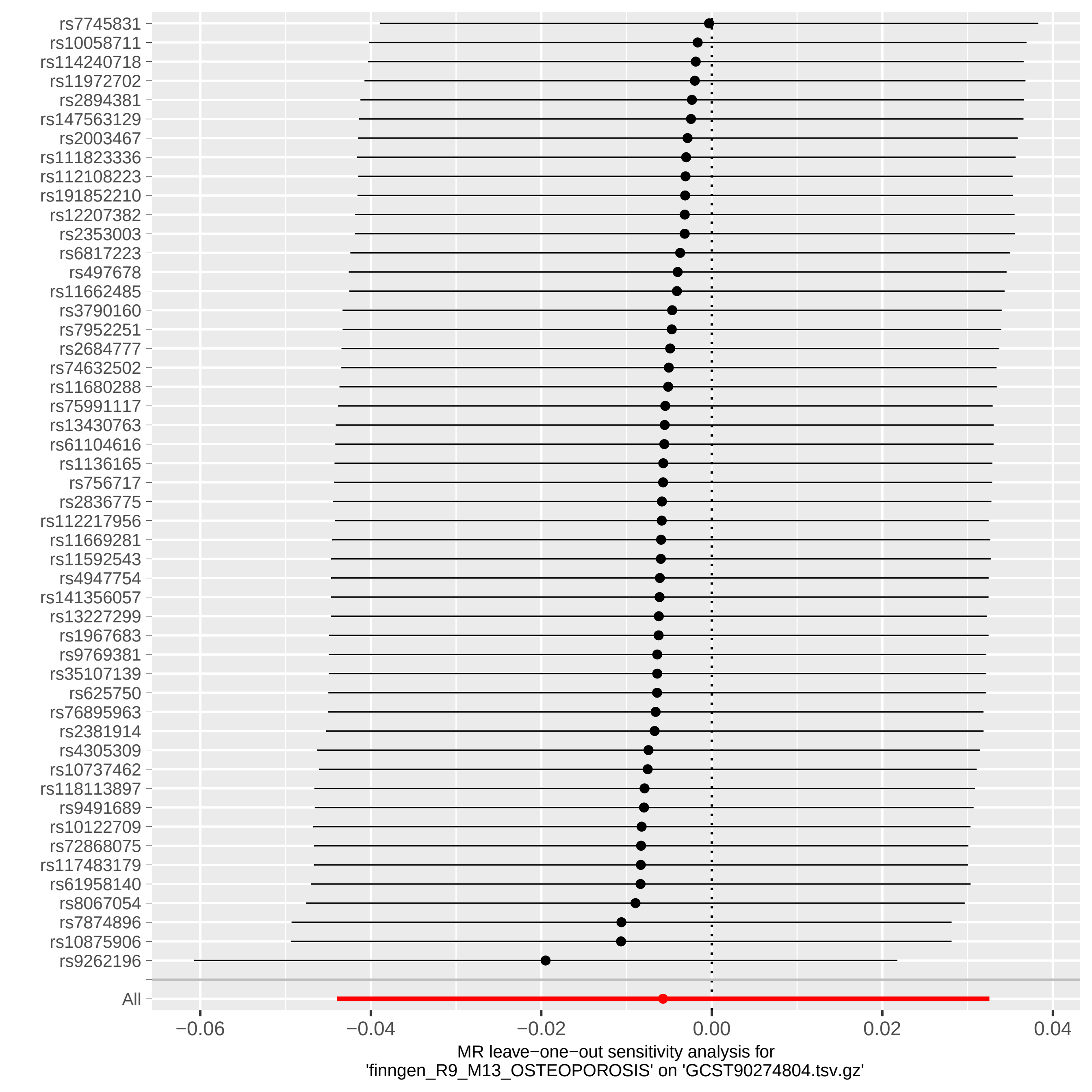
rs7745831
rs10058711
rs114240718
rs11972702
rs2894381
rs147563129
rs2003467
rs111823336
rs112108223
rs191852210
rs12207382
rs2353003
rs6817223
rs497678
rs11662485
rs3790160
rs7952251
rs2684777
rs74632502
rs11680288
rs75991117
rs13430763
rs61104616
rs1136165
rs756717
rs2836775
rs112217956
rs11669281
rs11592543
rs4947754
rs141356057
rs13227299
rs1967683
rs9769381
rs35107139
rs625750
rs76895963
rs2381914
rs4305309
rs10737462
rs118113897
rs9491689
rs10122709
rs72868075
rs117483179
rs61958140
rs8067054
rs7874896
rs10875906
rs9262196
All
−0.06
−0.04
−0.02
0.00
0.02
0.04
MR leave−one−out sensitivity analysis for
'finngen_R9_M13_OSTEOPOROSIS' on 'GCST90274804.tsv.gz'
